# Effect of Orbicularis Oculi Muscle Suspension Combined With Overlap in Modified Sub‐Brow Blepharoplasty: A Prospective Study

**DOI:** 10.1111/jocd.70103

**Published:** 2025-03-10

**Authors:** Jiangbo Cui, Yu Zhang, Zhengqiang Cang, Chaohua Liu, Jiao Cao, Zhaoxiang Zhang, Xing Fan, Xi Zhang, Baoqiang Song, Yang Li

**Affiliations:** ^1^ Department of Plastic Surgery, Xijing Hospital Fourth Military Medical University Xi'an Shaanxi China

## Abstract

**Background:**

Sub‐brow blepharoplasty (SBB) has emerged as one of the primary methods for addressing periorbital aging among East Asian women in recent years. Numerous surgeons have continued to refine and innovate this technique; however, challenges remain in maintaining the stability of surgical outcomes and addressing issues such as abnormal postoperative eyelid wrinkles.

**Objective:**

This study aims to conduct a prospective clinical investigation to demonstrate that improvements in SBB techniques can lead to enhanced postoperative eyelid morphology and sustained long‐term results.

**Method:**

Between September 2021 and September 2022, we included 72 female patients who underwent the modified SBB procedure. In addition to recording baseline data prior to surgery, we evaluated surgical outcomes postoperatively using both subjective and objective items.

**Result:**

The average age of the patients was 47.69 years, with a median grade of 2 for upper eyelid skin laxity. The mean length of excised tissue was 44.72 mm, while the average width was 9.66 mm. No significant differences were observed in the vertical distance from the superior brow edge to the center of the pupil at 6 and 12 months postoperation, measured at the inner edge of the iris, the center of the pupil, and the lateral canthus. Similarly, the GAIS scores showed no significant differences at 6 and 12 months postoperation.

**Conclusion:**

In this study, the improved SBB technique successfully reduced or eliminated abnormal postoperative eyelid creases, ensuring stable outcomes and an aesthetically pleasing appearance.

## 1 Introduction

The periorbital region is pivotal in facial aesthetics, profoundly affecting attractiveness and emotional expression [1]. However, it is among the first areas to show signs of aging, especially in the upper eyelids. Aging in the periorbital region often manifests as sagging at the outer corners of the eyes, wrinkles, skin laxity, and poorly defined double eyelid crease [2]. Consequently, upper blepharoplasty aimed at addressing these concerns has consistently ranked among the most popular aesthetic procedures. The sub‐brow blepharoplasty (SBB) technique was first introduced by Parkes [3] et al. in 1976 and has gained widespread application, particularly among East Asian women. Due to the higher brow position, longer distance between the brow and eyes, and greater fat deposits in the orbital septum of East Asian women [4], as well as a propensity for the development of crow's feet with aging, SBB has a broader appeal in this demographic. A survey indicated that East Asian women are more concerned about periorbital aging than brow ptosis [5].

**FIGURE 1:**
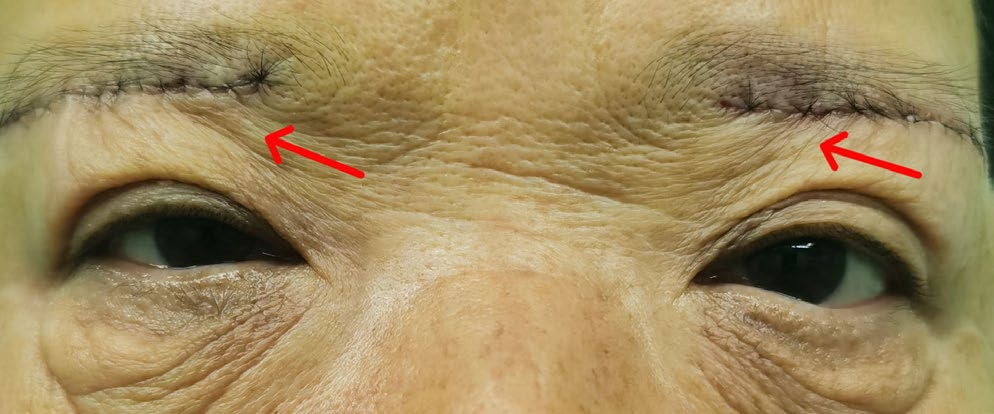
The red arrow indicates the abnormal directional lines on the upper eyelid following traditional SBB surgery.

Traditional sub‐brow blepharoplasty primarily entails the removal of lax skin through a sub‐brow incision. While this technique offers advantages such as minimal swelling and quick recovery, the tendency for recurrence poses a significant challenge. To optimize the SBB procedure, numerous scholars have proposed various innovations; however, some patients still experience suboptimal eyelid morphology and abnormal wrinkle directions postoperatively (Figure 1). Therefore, we aimed to further modify the SBB technique by building on previous clinical experiences to address issues of recurrence and undesirable aesthetics. In this study, we focus on the modified SBB procedure and seek to demonstrate the effectiveness of this improved technique through a prospective investigation.

## 2 Patient and Method

This study adhered to the Declaration of Helsinki and received approval from the ethics committee. All enrolled patients provided informed consent.

### 2.1 Patients Enrollment

The inclusion criteria for patients were as follows: (1) surgery was performed between September 2021 and September 2022; (2) patients were aged between 25 and 65 years; and (3) patients exhibited significant upper eyelid skin laxity that affected periorbital morphology or obstructed their visual field. The exclusion criteria were as follows: (1) patients with insufficient levator muscle strength or other chronic diseases that could affect the surgery; (2) patients with a history of other ocular cosmetic procedures; and (3) patients with incomplete clinical data or who withdrew from the study.

### 2.2 Preoperative Design

**FIGURE 2:**
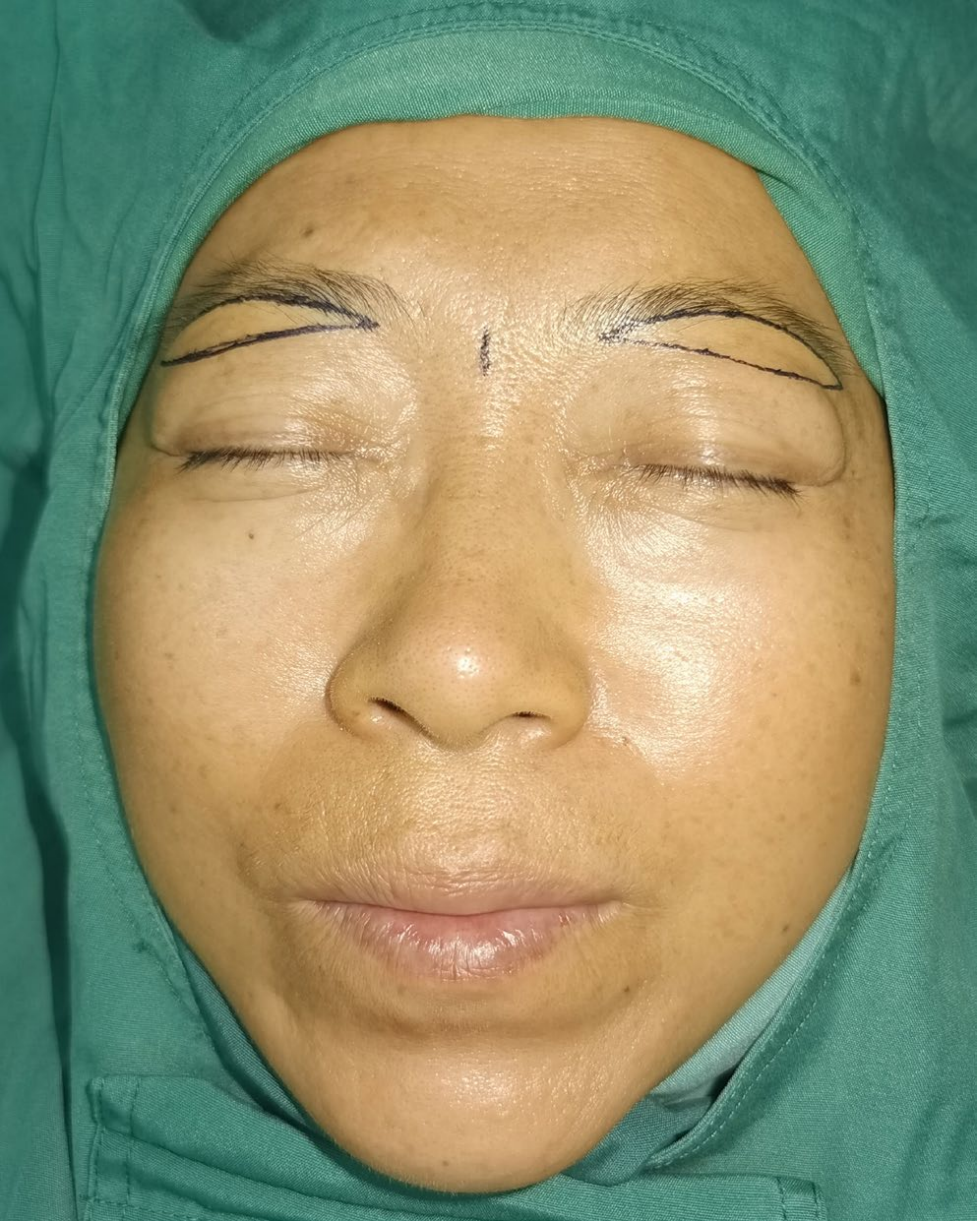
Preoperative design.

Prior to the procedure, patients were positioned supine. The surgeon used forceps to assess the amount of skin to be excised from the sub‐brow area, ensuring that excessive removal did not compromise eyelid closure. The upper incision began approximately 5 mm from the medial brow, following the lower brow contour laterally and terminating about 5 mm from the brow tail to minimize visible scarring. The lower incision aligned with the upper incision line. After securing the skin with forceps, the excision amount was confirmed, and the lower incision line was marked. The distance between the upper and lower incisions typically reached its widest point at the vertical line of the brow peak, tapering to align with the lateral end of the upper incision, thus creating a natural arc. The excised skin between the incisions had a spindle shape, with lengths varying from 40 to 50 mm and widths from 8 to 12 mm. For patients with significant lateral brow droop or an unsatisfactory brow shape, the incision design included removal of part of the brow body and drooping brow tail to facilitate future eyebrow tattooing and achieve the desired shape (Figure 2).

### 2.3 Surgery Technique

**FIGURE 3:**
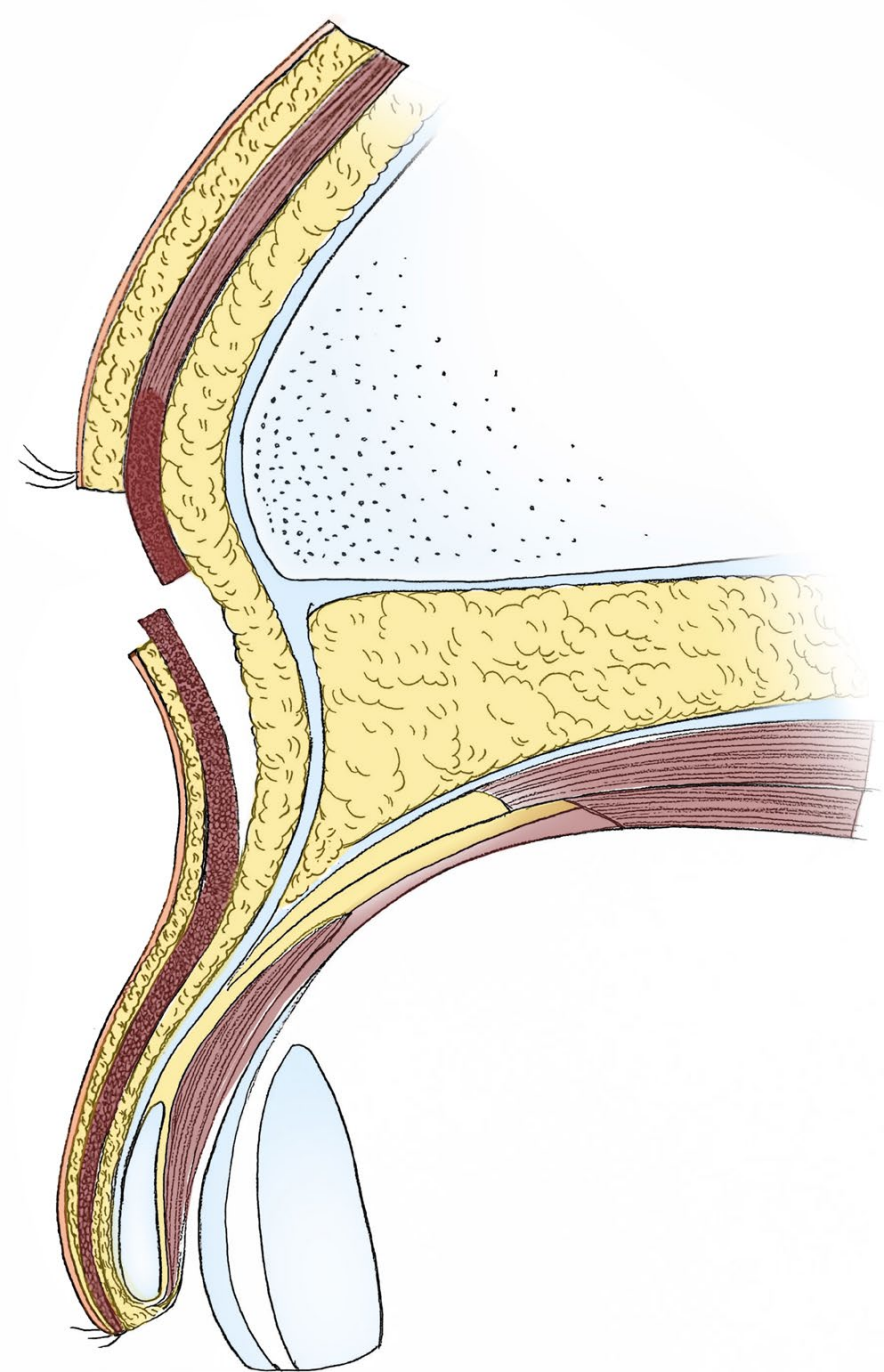
Dissection of the lower margin of the orbicularis oculi muscle at the surgical incision.

**FIGURE 4:**
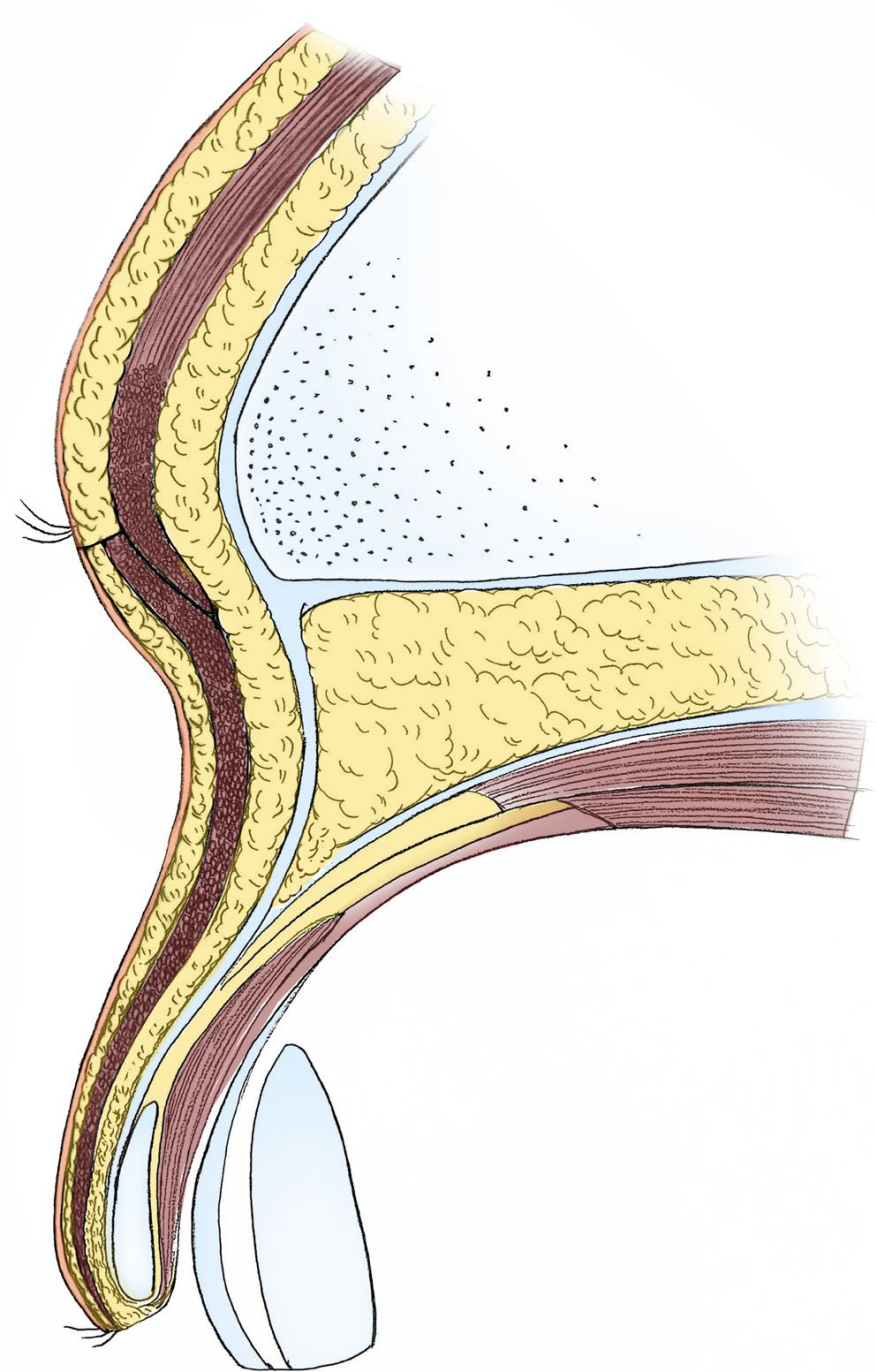
Suspension of the orbicularis oculi muscle at the lower margin of the surgical incision and overlapping suturing of the orbicularis oculi muscle at the upper margin of the incision.

All surgeries were performed by the same surgeon, and local anesthesia was employed using 2% lidocaine hydrochloride with 1:200000 epinephrine for infiltration of the surgical area. A #15 scalpel blade was then used to incise the skin and subcutaneous tissue along the marked line, ensuring the upper incision was parallel to the direction of the hair follicles to minimize damage to the eyebrows. The skin and subcutaneous fat within the marked area were removed to expose the orbicularis oculi muscle beneath the incision. Subsequently, the orbicularis oculi muscle was transected along the lower edge of the incision. Using a #15 scalpel blade, sharp dissection was performed between the orbicularis oculi muscle and its underlying fascia toward the eyelid margin, approximately 10 mm above the upper border of the double eyelid line, forming a muscle flap (Figure 3). The resulting muscle flap was then elevated to overlap with the orbicularis oculi muscle below the upper incision (forming an overlap of the muscle flaps) (Figure 4). The overlap of the orbicularis oculi muscle ensured a more uniform thickness of the upper and lower incisions, thereby preventing a stepped appearance. The muscle flap was secured to the upper incision edge with interrupted sutures using 5–0 absorbable thread, and the skin was closed with interrupted sutures using 7–0 cosmetic thread to achieve precise alignment of the incision edges. Antibiotic ointment was applied at the incision site, which was then covered with sterile gauze. Oral antibiotics were prescribed for 3 days to prevent infection, and ice packs were used for the first 3 days postoperatively to reduce bruising and swelling. Dressings were changed on the third postoperative day, and sutures were removed on the seventh postoperative day.

### 2.4 Assessment Item

**FIGURE 5:**
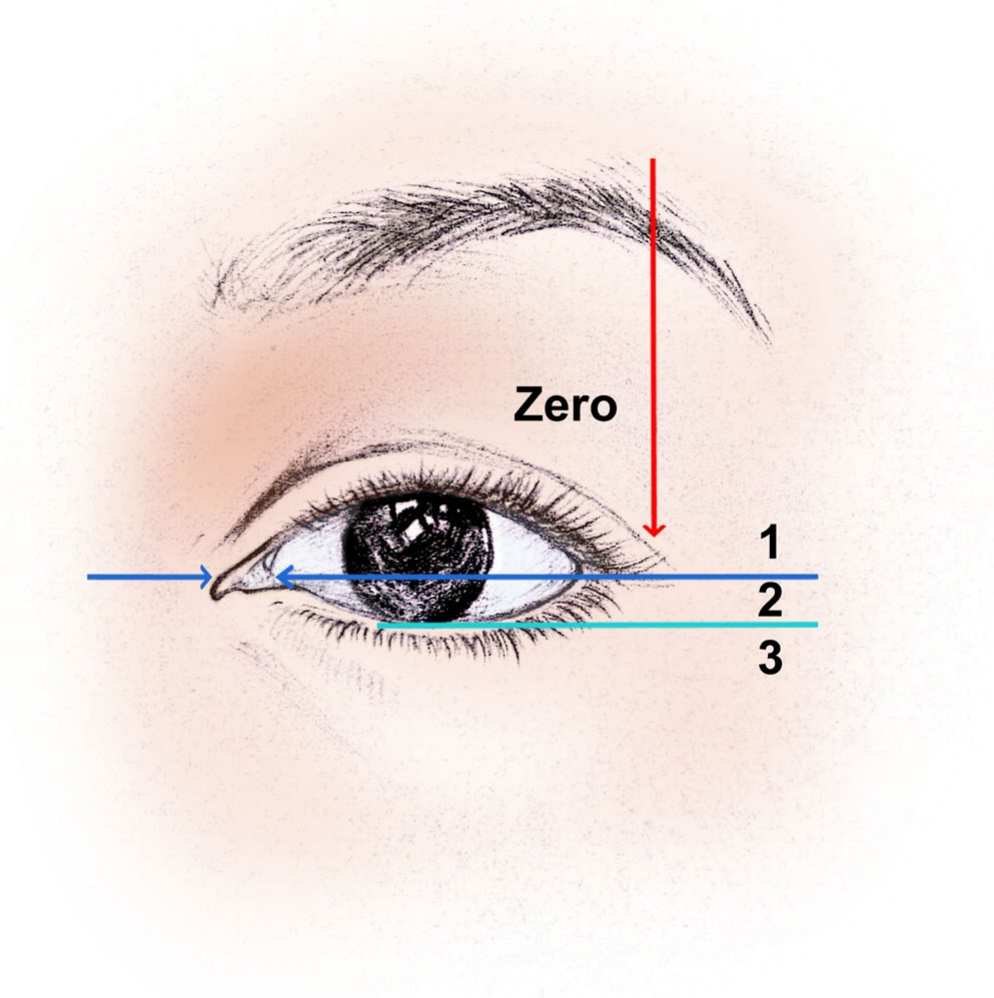
Illustrative diagram of upper eyelid skin laxity classification.

**FIGURE 6:**
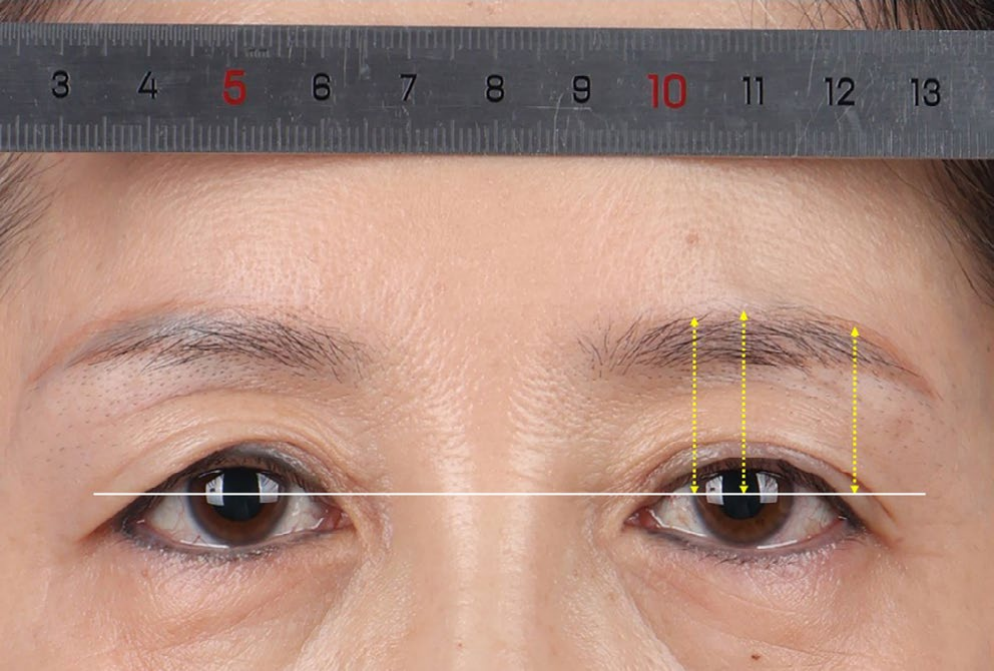
Illustrative diagram measuring the vertical distance from the upper eyebrow edge to the medial limbus, mid‐pupil, and lateral canthus postoperatively.

The data we require includes: (1) preoperative baseline information such as age and classification of upper eyelid skin laxity [6] [Grade 0: no skin laxity evident at the lateral aspect of the orbit; Grade 1: lax skin lower edge (LED) located above the intersection of the tear crest and upper eyelid margin; Grade 2: LED between the intersection of the tear crest and upper eyelid margin and above the lower margin of the iris; and Grade 3: LED below the lower margin of the iris (Figure 5)], as well as the length and width of the excised tissue; (2) objective assessment of the change in the vertical distance from the superior brow margin to the medial limbus, the midpoint of the pupil, and the lateral canthus at 6 and 12 months postoperatively compared to preoperative measurements (Figure 6); (3) objective evaluation of any abnormal skin lines on the upper eyelid postoperatively; (4) subjective assessment through the Global Aesthetic Improvement Scale (GAIS) [7] ratings by both the patient and another fixed physician for standardized photographs taken at 6 and 12 months postoperatively (−1 indicates deterioration, 0 indicates no change, 1 indicates slight improvement, 2 indicates significant improvement with room for adjustment, and 3 indicates complete improvement); and (5) postoperative complications.

### 2.5 Photo Evaluation

We utilized standardized preoperative and postoperative photographs of patients to evaluate surgical outcomes and conduct GAIS scoring. During the photo sessions, patients were positioned upright, looking straight ahead, and maintaining a level Frankfurt horizontal plane. Consistency was ensured in the patient's posture, expressions, and camera focus. The periorbital muscles of the patients were completely relaxed during photography. To preserve the original proportions, all photographs were uniformly modified using Adobe Photoshop CC (Adobe Systems Inc., San Jose, CA). Additionally, we enlisted other physicians to assess random photographs for an impartial evaluation of surgical results. Furthermore, the vertical distances from the superior brow margin to the medial limbus, the mid‐pupil, and the lateral canthus were also measured using standardized photographs.

### 2.6 Statistical Analysis

Data analysis was conducted using SPSS (IBM SPSS 26.0, IBM Corp., Armonk, NY, USA). For the changes in objective and subjective assessments at 6 and 12 months postoperative, we employed the Wilcoxon signed‐rank test. Results are presented as mean ± standard deviation, median, and interquartile range. A *p*‐value of less than 0.05 was considered to indicate a significant difference.

## 3 Results

From September 2021 to September 2022, we enrolled a total of 72 female patients, with ages ranging from 28 to 59 years and a mean age of 47.69 years. The median classification of upper eyelid skin laxity was Grade 2. The average length of excised tissue across all patients was 44.72 mm and the average width was 9.66 mm. Detailed baseline information for the patients is presented in Table 1.

**TABLE 1:** Patient demographics before surgery.

Characteristics	Value
Age at surgery, year	
Mean ± SD	47.69 ± 9.06
Range	28–59
Sex	
Male	0
Female	72
Grade of skin laxity [median (P25, P75)]	2 (2,2.75)
Size of excised tissue, mm (mean ± SD)	
Length	44.72 ± 4.62
Width	9.66 ± 2.16

For the changes in postoperative objective data, we measured the vertical distances from the superior brow margin to the medial limbus, mid‐pupil, and lateral canthus at 6 and 12 months postoperatively and compared the two sets of data. The results indicated that there were no significant differences in the vertical distances from the superior brow margin to the medial limbus, mid‐pupil, and lateral canthus between the 12‐ and 6‐month assessments (Table 2). We also recorded the incidence of abnormal skin lines on the upper eyelids postoperatively. Among the 72 patients, 8 presented with abnormal eyelid lines, including 1 patient with Grade 1 skin laxity, 4 patients with Grade 2, and 3 patients with Grade 3. Although only a small number of patients exhibited abnormal eyelid lines, the condition was most prevalent among those with Grade 3 skin laxity, potentially related to the larger volume of skin excised and the inherent liability of the patient's eyelid skin. However, all patients with abnormal eyelid lines had these lines resolved within 3 months postoperatively due to our folding suture technique (Table 3).

**TABLE 2:** Distance from superior brow edge to center of pupil.

	Medial limbus (mm)	Mid‐pupil (mm)	Lateral canthus (mm)
6 M post	23.24 ± 2.33	22.82 ± 2.58	23.64 ± 2.41
12 M post	23.73 ± 2.47	23.23 ± 2.64	24.11 ± 2.44
*p*	0.15	0.09	0.72

*Note:* The Wilcoxon signed‐rank test was used to compare vertical heights of three points at 6 and 12 months postoperatively, and *p* < 0.05 was considered statistically significant. The outcomes were expressed as mean ± SD.

**TABLE 3:** Postoperative abnormal eyelid contour.

Grade	Patient count	Abnormal eyelid contour	Time for contour resolution, month
1	12	1	1 m
2	42	4	1.63 m
3	18	3	2.5 m

Regarding GAIS scores, there were no significant differences observed between the physician‐assessed scores and patient self‐assessments at 6 and 12 months postoperative (Table 4).

**TABLE 4:** GAIS scores.

	6 Months postoperatively	12 Months postoperatively	*p*
Patient scores	2 (2,3)	2 (2,2)	0.118
Physician scores	2 (2,3)	2 (2,3)	0.306

*Note:* The Wilcoxon signed‐rank test was used to compare GAIS scores at 6 and 12 months postoperatively, and *p* < 0.05 was considered statistically significant. The outcomes were expressed as median (P25, P75).

We also recorded postoperative complications across all patients (Table 5). In the short term, four patients experienced frontal numbness, and six experienced nausea and vomiting, likely due to manipulation of the supraorbital or trochlear nerves during surgery, both of which resolved shortly after. One patient developed a wound infection due to inadequate self‐care, which was promptly addressed. Five patients experienced bruising, with one patient having a severe hematoma that required surgical intervention on the 2nd day postoperatively. Additionally, one patient exhibited noticeable bilateral periorbital asymmetry, which was corrected with two surgical procedures. No patients developed hypertrophic scars postoperatively.

**TABLE 5:** Postoperative complications.

Complications	Number of cases
Infection	1
Congestion	5
Scar hyperplasia	0
Asymmetry	1
Forehead numbness	4
Nausea	6

## 4 Cases Report

### 4.1 Case 1

**FIGURE 7:**
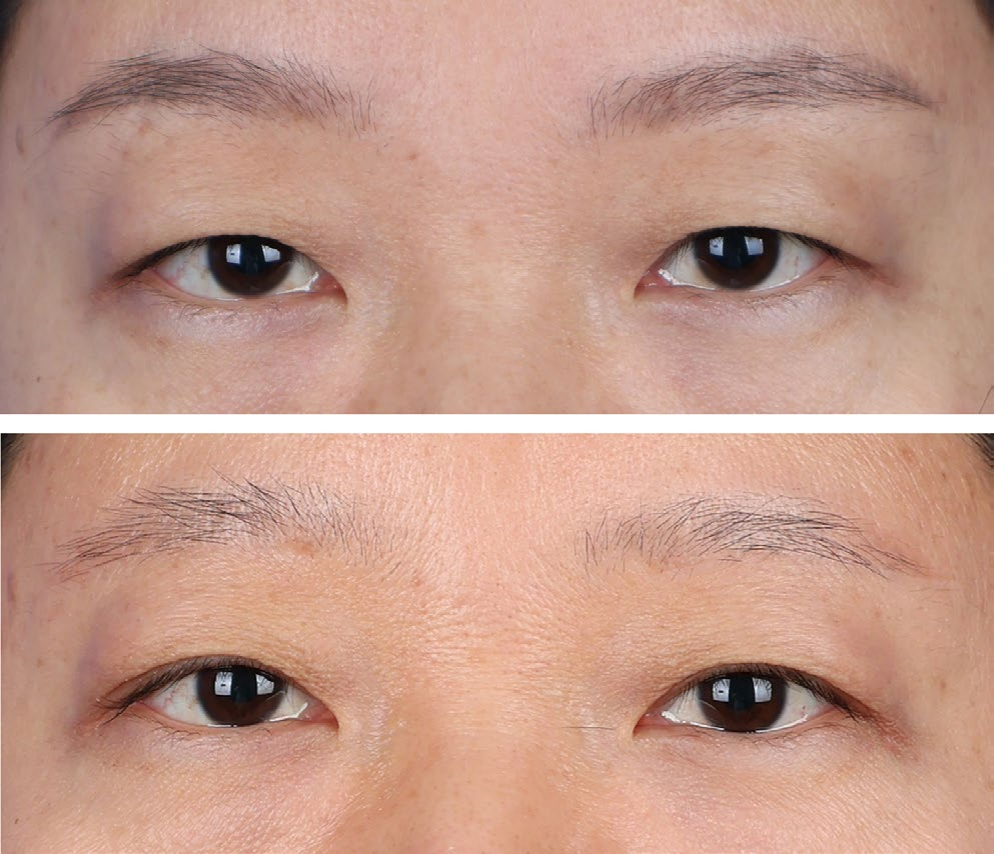
Comparative photographs of Case 1 pre‐ and postoperatively; the images reveal that 1 year after surgery, the patient exhibits satisfactory results, with no abnormal lines on the upper eyelid and the double eyelid folds fully visible.

The patient is a 41‐year‐old female with a preoperative upper eyelid skin laxity classified as Grade 1. She had not undergone any prior oculoplastic surgeries. During the procedure, the excised tissue length from her upper eyelid measured 43 mm, and the width was 7.5 mm. At 6 and 12 months postoperatively, there were minimal changes in the vertical distances from the superior brow margin to the medial limbus, mid‐pupil, and lateral canthus, indicating stable surgical outcomes. Furthermore, the patient did not experience any abnormal skin lines on the upper eyelid postoperatively and expressed high satisfaction with the results (Figure 7).

### 4.2 Case 2

**FIGURE 8:**
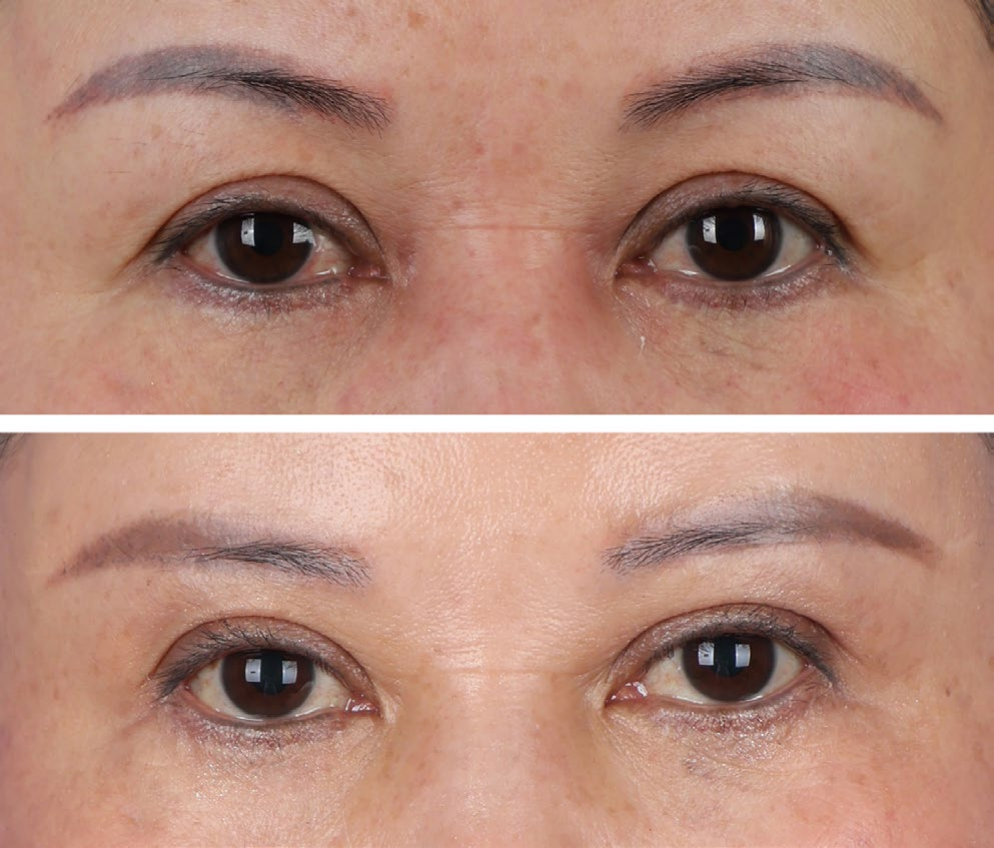
Comparative photographs of Case 2 pre‐ and postoperatively; the images show that 1 year after surgery, the patient has no abnormal lines on the upper eyelid, significant improvement in periorbital aging, and the double eyelid folds appear naturally aesthetic.

The patient is a 51‐year‐old female with a preoperative upper eyelid skin laxity classified as Grade 2. She had not previously undergone any oculoplastic surgeries. During the procedure, the excised tissue length from her upper eyelid measured 47 mm, and the width was 11 mm. The patient experienced periorbital bruising postoperatively, which resolved quickly. At 6 and 12 months postoperatively, there were almost no changes in the vertical distances from the superior brow margin to the medial limbus, mid‐pupil, and lateral canthus, demonstrating excellent maintenance of surgical outcomes. Additionally, the patient did not develop any abnormal skin lines on the upper eyelid postoperatively and expressed high satisfaction with the results (Figure 8).

## 5 Discussion

The periorbital region, which includes the eyes, eyelids, and eyebrows, is a crucial area for facial recognition, emotional expression, and attractiveness. The characteristics of the periorbital region vary across different ethnicities and races. For example, Asian individuals typically exhibit fuller upper eyelids, thicker eyelid skin, and higher eyebrows, making them more suited for sub‐brow blepharoplasty (SBB) [8]. In contrast, individuals of European descent have more prominent upper orbital rims, relatively lower eyebrows, less retro‐orbital fat, and thinner upper eyelid skin [9]. This ethnic difference in eyebrow height suggests that SBB is less appropriate for Europeans, with a brow lift being a more suitable option.

Initially, the sub‐brow incision blepharoplasty primarily involved the excision of the upper eyelid skin to correct upper eyelid ptosis [4]; however, this approach was prone to recurrence. Kim YS [10] and Qiu Y [11] removed the relaxed orbital orbicularis oculi muscle and sutured the muscle remnants to correct severe upper eyelid laxity, achieving long‐term postoperative results. Ichinose A [12] and colleagues, in their sub‐brow incision blepharoplasty, excised excess skin without removing the orbicularis oculi muscle, securing the muscle to the subcutaneous tissue at the incision's upper edge to achieve eyelid lift and fixation. Wang J [13] and Kim YS [14] fixed the orbicularis oculi muscle to the periosteum at the supraorbital margin, performing a suspension technique to correct upper eyelid laxity while alleviating periorbital wrinkles. Kim HS [15] and colleagues suspended the orbicularis oculi muscle at the frontalis muscle level to preserve the brow's sliding plane, allowing for natural brow movement and avoiding potential damage to the deep branch of the supraorbital nerve from fixation to the periosteum.

Aging in the periorbital region is the result of multifactorial changes, including alterations in skin, fat, muscle, and ligaments, all of which contribute to the aging process [16]. Therefore, addressing only one factor is insufficient to fully resolve periorbital aging, and patients may experience secondary descent postoperatively. We have recognized these issues in our clinical practice, and as our understanding of periorbital anatomy and aging has deepened, our department has made a series of improvements and innovations to the SBB technique. These include bisecting the orbicularis oculi muscle flap and cross‐fixing it to the periosteum to maintain long‐lasting lateral canthus elevation [13]; inverting and folding the eyebrow fat pad to treat mild‐to‐moderate upper eyelid hollowness [17]; and combining granular fat injection with brow lift techniques for severe upper eyelid hollowness [18]. These improvements have yielded positive postoperative outcomes.

In this study, we employed a technique that combines the elevation of the orbicularis oculi muscle flap with overlapping sutures, facilitating a stable lifting effect through scar adhesions formed between the muscle layers. Furthermore, we believe that preserving the orbicularis oculi muscle at the lower edge of the incision is a more appropriate choice. Firstly, maintaining the orbicularis oculi in this area ensures a sufficient overlapping surface area between the muscles at the upper and lower edges of the incision, thereby enhancing the adhesion strength and ensuring the stability and reliability of the muscle suspension. Secondly, this approach promotes a more uniform tissue thickness at the incision margins, preventing a noticeable step‐off effect and achieving a natural transition in tissue thickness from the upper eyelid to the brow. At 6 and 12 months postoperatively, there were no significant changes in the vertical distances from the superior brow margin to the medial limbus, mid‐pupil, and lateral canthus, indicating no notable eyebrow descent and confirming that our technical enhancements can ensure stable postoperative results. Additionally, since the maximum excision of eyelid skin occurs laterally, traditional surgical methods often lead to abnormal vertical wrinkles along the eyelid margin due to increased tension on the skin after suturing the upper and lower incisions. Our modified technique mitigates this by releasing the fibrous connections between the eyelid skin and deeper tissues through dissection between the orbicularis oculi muscle and deep fascia, enhancing the extensibility and compliance of the upper eyelid skin flap. This effectively disperses the tension concentrated on the upper eyelid's medial and lateral aspects across the entire eyelid, thereby avoiding or alleviating abnormal skin lines.

Moreover, in previous SBB techniques, discrepancies in the thickness of the skin and muscle along the upper and lower cutting edges often resulted in poor incision alignment and a stepped appearance. Our modified technique, which involves overlapping muscle layers, increases the thickness at the incision's lower edge, facilitating precise skin approximation and yielding a flatter incision postsuturing. The strong adhesion between the muscle layers effectively reduces wound tension and minimizes scar hypertrophy. Additionally, during the procedure, we made a transverse incision along the lower edge of the orbicularis oculi muscle, disrupting part of the circular muscle bundles without affecting eyelid closure function. This approach reduces muscle contraction strength, thereby alleviating local tension on the upper eyelid and lessening the crow's feet caused by orbicularis oculi muscle contractions at the outer corner of the eye. In clinical practice, we can further optimize outcomes for patients with varying conditions by combining our improved technique with approaches such as the removal or repositioning of orbital fat and the dissection of the corrugator supercilii muscle.

Our study has certain limitations. First, although a substantial number of patients were included, we did not conduct comparative studies. A comparative analysis could provide more persuasive evidence regarding the impact of our surgical technique improvements on postoperative results. Second, our follow‐up duration was limited to 12 months. Extending the follow‐up period to 24 months or longer would likely better demonstrate the long‐term effects of our surgical outcomes.

## 6 Conclusion

In suprabrow blepharoplasty, we utilized a technique that combines orbicularis oculi muscle suspension with overlapping sutures. This approach mitigated or eliminated abnormal skin lines on the upper eyelid postoperatively, ensuring stable outcomes and favorable results.

## Author Contributions

J.C., Y.Z., Z.C., X.F., and X.Z. performed the research. Y.L. and B.S. supervised the research study. C.L., J.C., and Z.Z. analyzed the data. J.C. and Y.Z. wrote the paper.

## Ethics Statement

This study received approval from the Ethics Committee of Xijing Hospital, with the approval number KY20212014‐F‐1.

## Conflicts of Interest

The authors declare no conflicts of interest.

## Supporting information


**Video S1.** Surgical video: Demonstrating orbicularis oculi muscle preservation technique in modified sub‐brow blepharoplasty.

## Data Availability

The data that support the findings of this study are available from the corresponding author upon reasonable request.
